# Supplementary figures and images for: Insights into the Flavor Differentiation between Two Wild Edible Boletus Species through Metabolomic and Transcriptomic Analyses
Source: Foods. 2023 Jul 18;12(14):2728. doi: 10.3390/foods12142728 (PMC10380016; doi:10.3390/foods12142728)

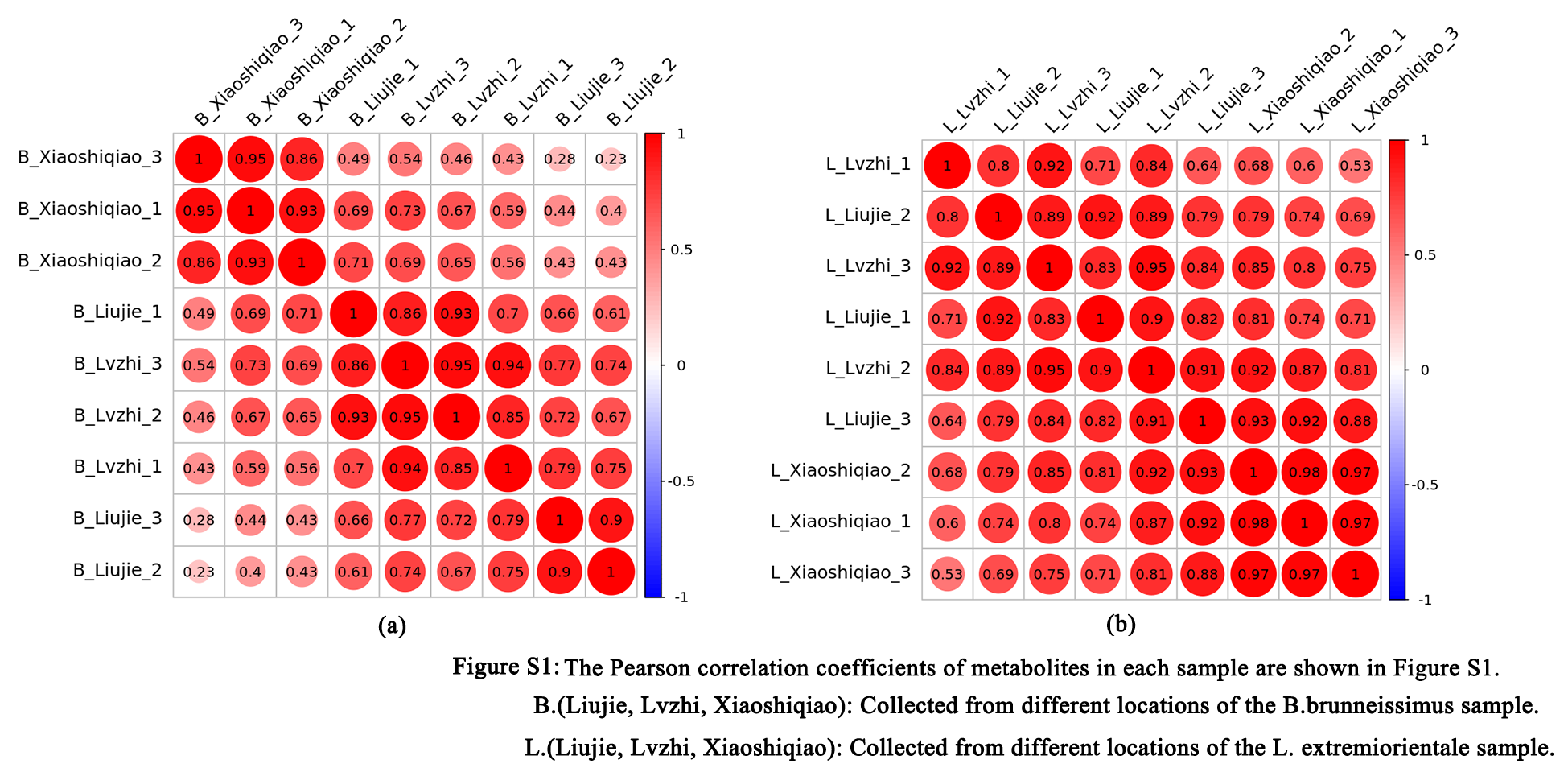

Supplement: Supplementary file 1 [file foods-12-02728-s001.zip › Figure S/Figure S1.tif]

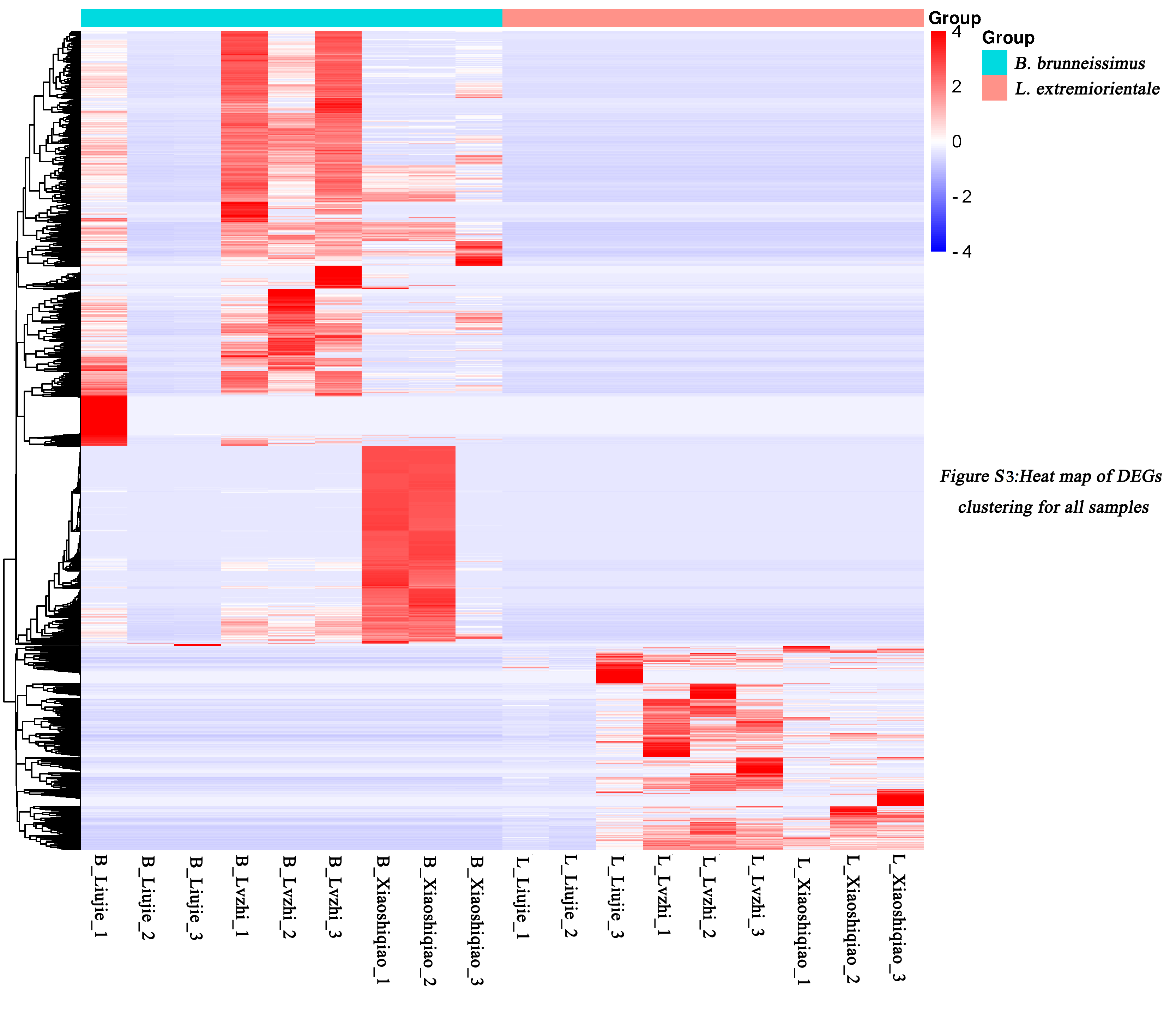

Supplement: Supplementary file 1 [file foods-12-02728-s001.zip › Figure S/Figure S3.tif]

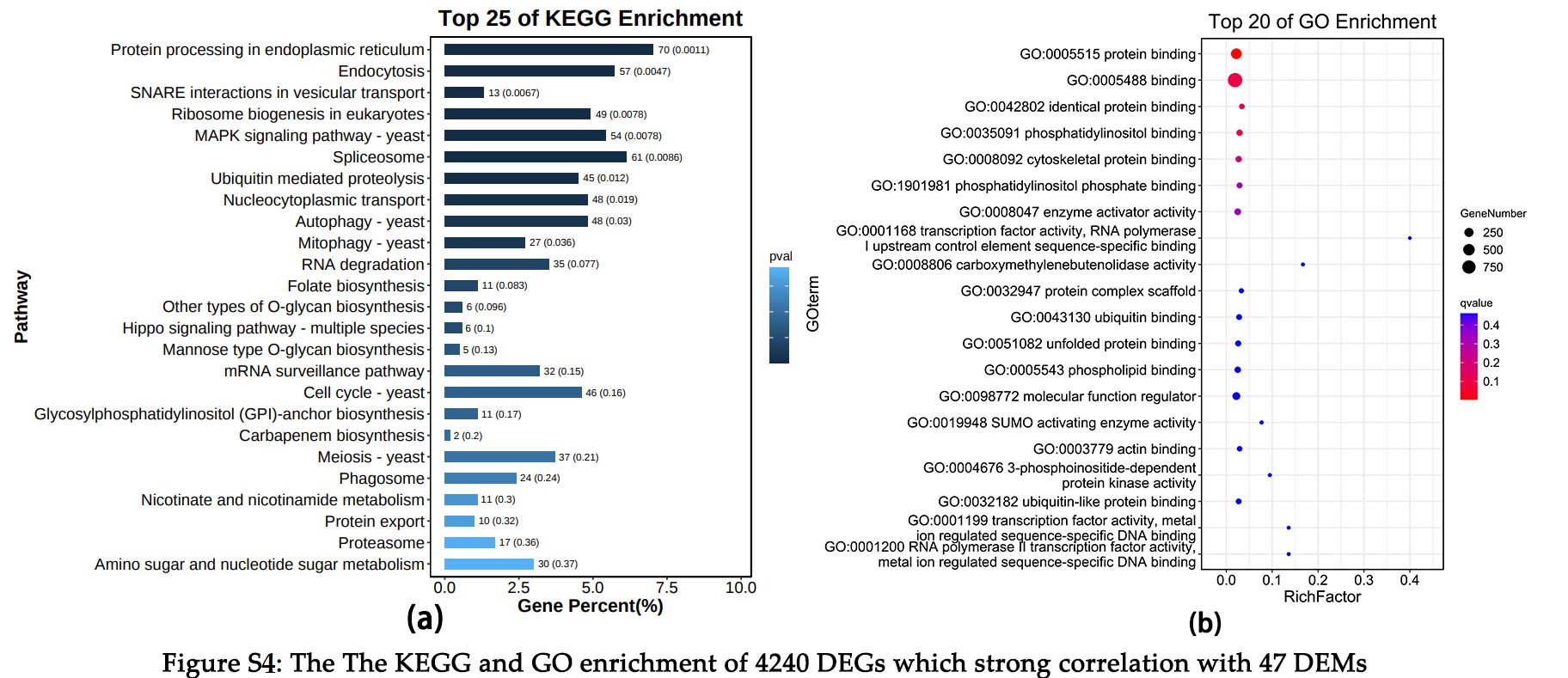

Supplement: Supplementary file 1 [file foods-12-02728-s001.zip › Figure S/Figure S4.jpg]
